# Biomarker-Oriented Therapy in Bladder and Renal Cancer

**DOI:** 10.3390/ijms22062832

**Published:** 2021-03-11

**Authors:** Mathijs P. Scholtes, Arnout R. Alberts, Iris G. Iflé, Paul C. M. S. Verhagen, Astrid A. M. van der Veldt, Tahlita C. M. Zuiverloon

**Affiliations:** 1 Department of Urology, Erasmus MC Cancer Institute, University Medical Center Rotterdam, 3015 GD Rotterdam, The Netherlands; m.scholtes@erasmusmc.nl (M.P.S.); a.r.alberts@erasmusmc.nl (A.R.A.); 407644ii@student.eur.nl (I.G.I.); p.verhagen@erasmusmc.nl (P.C.M.S.V.); 2 Department of Oncology, Erasmus MC Cancer Institute, University Medical Center Rotterdam, 3015 GD Rotterdam, The Netherlands; a.vanderveldt@erasmusmc.nl; 3 Department of Radiology & Nuclear Medicine, Erasmus MC Cancer Institute, University Medical Center Rotterdam, 3015 GD Rotterdam, The Netherlands

**Keywords:** biomarker guided therapy, targeted therapy, bladder cancer, renal cancer, immune checkpoint inhibitor

## Abstract

Treatment of patients with urothelial carcinoma (UC) of the bladder or renal cancer has changed significantly during recent years and efforts towards biomarker-directed therapy are being investigated. Immune checkpoint inhibition (ICI) or fibroblast growth factor receptor (FGFR) directed therapy are being evaluated for non-muscle invasive bladder cancer (NMIBC) patients, as well as muscle-invasive bladder cancer (MIBC) patients. Meanwhile, efforts to predict tumor response to neoadjuvant chemotherapy (NAC) are still ongoing, and genomic biomarkers are being evaluated in prospective clinical trials. Currently, patients with metastatic UC (mUC) are usually treated with second-line ICI, while cisplatin-ineligible patients with programmed death-ligand 1 (PD-L1) positive tumors can benefit from first-line ICI. Platinum-relapsed UC patients harboring FGFR2/3 mutations can be treated with erdafitinib, while enfortumab vedotin has emerged as a novel third-line treatment option for mUC. In metastatic (clear cell) renal cell carcinoma (RCC), ICI was first introduced as second-line treatment after vascular endothelial growth factor receptor—tyrosine kinase inhibition (VEGFR-TKI). Currently, ICIs have also been introduced as first-line treatment in metastatic RCC. Although there is no evidence up to now for beneficial adjuvant treatment after surgery with VEGFR-TKIs in high-risk non-metastatic RCC, several trials are underway investigating the potential beneficial effect of ICIs in this setting.

## 1. Introduction

Bladder cancer and renal cancer are among the 10 most common cancers worldwide. In recent years, significant progress was made in terms of biomarker-oriented treatment for both urogenital cancer types. Here, we provide a comprehensive overview of new developments in targeted therapy for urothelial carcinoma of the bladder and renal carcinoma.

## 2. Bladder Cancer

Over 400,000 patients are newly diagnosed with bladder cancer (BC) every year [1]. Most patients with BC are diagnosed with urothelial carcinoma (UC) of the bladder, although urothelial carcinoma can also develop in the ureter or renal pelvis, which is generally referred to as upper tract urothelial carcinoma (UTUC). Three quarters of BC patients have tumors that do not invade the detrusor muscle, which is described as non-muscle invasive bladder cancer (NMIBC). NMIBC is characterized by a relatively good prognosis, allowing most NMIBC patients to undergo only local treatment to prevent tumor recurrence and progression to muscle-invasive bladder cancer (MIBC). MIBC is characterized by worse prognosis due to its nature of developing early metastasis. Therefore, MIBC patients are treated with neoadjuvant chemotherapy followed by surgical removal of the bladder (radical cystectomy) and pelvic lymph node dissection [2]. Despite this intensive treatment, approximately half of the MIBC patients will develop metastatic disease. Metastatic urothelial carcinoma (mUC) is treated in a palliative setting with platinum-based chemotherapy regimens or immune checkpoint inhibition (ICI) in case a patient is platinum-unfit [2].

## 3. Non-Muscle Invasive Bladder Cancer (NMIBC)

Treatment of NMIBC patients is based on the risk of tumor recurrence and/or progression [3]. Patients with high-risk NMIBC are treated with transurethral resection of bladder tumors (TURBT) followed by Bacille Calmette Guerin (BCG) instillations up to three years [3]. Standard treatment for patients with tumors that recur after adequate BCG treatment (high-risk, BCG-unresponsive NMIBC patients) is surgical removal of the bladder (cystectomy) with urinary diversion [3]. A cystectomy is associated a high risk of complications and mortality (1–2% dies within 30 days), and a decreased patients’ quality of life [4,5]. There is currently no standard treatment for patients who are ineligible for, or refuse a cystectomy. Thus, ongoing research aims to provide alternative treatment options for high-risk BCG-unresponsive NMIBC patients (Table 1).

As of January 2020, the United States Food and Drug Administration (FDA) approved immune checkpoint inhibitor pembrolizumab (anti-PD-1) for treating high-risk BCG-unresponsive NMIBC patients. However, no biomarkers predictive of response to ICI have thus far been reported for NMIBC. FDA-approval was based on preliminary results of the KEYNOTE-057 trial (NCT02625961) [6,7]. This phase II trial reported promising 3-month complete response rates (CRR) in 44.6% of patients with carcinoma in situ (CIS) (N = 65), and 41.7% of patients with T1 tumors and concomitant CIS (N = 12) [7]. Interestingly, observed responses were durable with 52.6% of complete responses (CR) lasting over 12 months, and most importantly, no progression to MIBC or mUC was observed [7]. Preliminary analysis of a comparable phase II trial (SWOG S1605) which is evaluating PD-L1 inhibitor atezolizumab showed a 3-month CRR of 41.1% for patients with Ta/T1 tumors and concomitant CIS (N = 30) (NCT02844816) [8]. The SWOG S1605 trial will also investigate whether PD-L1 and CD8 expression assessed by immunohistochemistry (IHC), and immune signatures determined by RNA sequencing can predict response to atezolizumab. In short, preliminary reports of phase II trials imply that ICI might present as an alternative to a cystectomy for BCG-unresponsive high-risk NMIBC patients, although, biomarkers predictive of response are currently lacking. Additionally, longer follow-up is awaited in order to evaluate the longevity of CRRs before we learn whether ICI can postpone or avert a radical cystectomy in NMIBC patients.

NMIBC is molecularly characterized by activating fibroblast growth factor receptor 3 (FGFR3) mutations, which are present in 20–80% of patients, depending on tumor stage [9]. FGFR3 is a receptor tyrosine kinase that mutates to become constitutively active and drive tumor growth through activation of downstream MAPK and/or Pi3K pathway signaling [9,10,11]. It has been hypothesized that BC patients with somatic FGFR3 mutations would benefit from FGFR3 inhibition, and currently two FGFR-inhibitors are under investigation for treating NMIBC patients. A phase I study investigated the safety of 125mg infigratinib, a selective FGFR1-3 inhibitor, in BCG-unresponsive NMIBC patients with FGFR3 mutations or gene-fusions, but the trial was terminated due to toxicity (including eye, skin and nail toxicities) (NCT02657486) [12]. Noteworthy, three out of four, FGFR3-mutated, BCG-unresponsive patients demonstrated a CR determined by cystoscopy and cytology at 7 weeks of follow-up [12]. FGFR1-4 inhibitor erdafitinib is already FDA-approved for treating mUC patients, and preliminary efficacy and safety is now being evaluated in a phase II trial enrolling NMIBC patients with somatic FGFR3-mutations or fusions (NCT04172675) [13]. Patients with either high-risk BCG-unresponsive NMIBC (with or without CIS), or intermediate-risk NMIBC patients without prior intravesical treatment will be enrolled for 8–9 mg orally dosed erdafitinib.

Besides FGFR inhibitors, other tyrosine kinase inhibitors have been investigated. Dovitinib is a multikinase inhibitor targeting members of the RTK superfamily, including FGFRs, vascular endothelial growth factor receptor (VEGFR), platelet-derived growth factor receptor (PDGFR), and stem cell factor receptor (c-KIT). Oral dovitinib 500mg was investigated in a phase II trial (N = 13) enrolling BCG-unresponsive NMIBC patients harboring somatic FGFR3 mutations determined by SNaPshot mutations analysis and/or increased expression phosphorylated FGFR3 (pFGFR3) assessed by IHC. Only patients with both FGFR3 mutations and increased expression of pFGFR3 showed a CR at six months (33%), whereas patients with only pFGFR3 expression did not demonstrate any tumor response [14]. Lack of clinical activity in patients with pFGFR3 over-expressing tumors suggests that pFGFR3-based selection does not enrich for patients who benefit from dovitinib, and potentially FGFR3-inhibition in general. Sunitinib is another multikinase inhibitor being evaluated in NMIBC patients, albeit no biomarkers that might predict response to sunitinib are being evaluated. A phase II trial investigated 12-weeks of sunitinib in BCG-refractory NMIBC patients (N = 19) [15]. Although sunitinib was considered safe, observed clinical benefit was not demonstrated and the trial was terminated early, concluding with a 12 month progression free survival of 22%, which in this patient population is equal to no treatment at all. Another phase II trial investigated six weeks of induction BCG subsided by 28 days of sunitinib PO for high-risk BCG-naïve NMIBC patients [16]. The reported three-month CRR was 72%, whereby responders exhibited a significant increase in urinary VEGF-D levels after sunitinib treatment [16]. These observations warrant future studies investigating the role in VEGF in BC tumorigenesis and possibilities for sunitinib + BCG combination therapy. In conclusion, novel treatment options are emerging for high-risk BCG-unresponsive and BCG-naïve NMIBC patients ineligible for, or declining cystectomy. ICI demonstrated promising 6-month CRR for high-risk BCG-unresponsive patients, leading to FDA-approval of pembrolizumab. Nonetheless, 12-month PFS and CRS are awaited, and predictive biomarkers remain to be evaluated. Erdafitinib is already FDA-approved for mUC patients with FGFR3 mutations or gene-fusions, and is currently under investigation in NMIBC patients as alternative to radical cystectomy. Meanwhile, patient selection based on pFGFR3 over-expression does not enrich for response to anti-FGFR therapy. Whereas urinary VEGF-D kinetics seem to be associated with response to treatment with BCG followed by sunitinib, warranting further investigations.

## 4. Muscle Invasive Bladder Cancer (MIBC)

Standard of care for treating MIBC is a radical cystectomy with or without cisplatin-based neoadjuvant chemotherapy (NAC) [2]. In patients with cT2-3 MIBC, addition of NAC increases survival rates from approximately 45% to 50% [17,18]. During the last couple of years, efforts were put into prospectively identifying patients who will benefit from NAC. Comparison of survival data of MIBC patients with aberrant differentiation patterns showed that patients with small cell neuroendocrine tumors gain survival benefit when treated with NAC [2,19]. In contrast, patients with pure squamous cell carcinoma (SCC), a non-UC histological variant, do not benefit from NAC [20].

Other studies investigated tumor response to NAC in association with specific somatic mutations. Descriptive genomic analysis of pre-NAC tumor samples identified mutations in ERBB2 and DNA repair genes; ATM, RB1, FANCC, and ERCC2 were associated with tumor response to NAC [21,22,23,24]. Currently two clinical trials are evaluating biomarker potential of the aforementioned DNA-repair genes to predict NAC-response. The RETAIN trial is a phase II trial investigating whether patients with ATM, RB1, FANC and/or ERCC2 mutations can be safely monitored by active surveillance after TURBT-confirmed complete NAC-response (pT0), as an alternative for the standard of care radical cystectomy or chemoradiotherapy (NCT02710734) [25]. Meanwhile, another phase II study evaluates whether patients with mutated DNA-repair genes and non-invasive downstaging (cT0, cTa, cTis) after NAC can be treated with bladder sparing therapy instead of radical cystectomy or chemoradiotherapy (NCT03609216).

RNA-based gene-expression profiling has also been used to characterize MIBC tumors and associate particular gene-expression patterns with tumor response to NAC. Gene-expression profiling efforts by independent groups have identified several MIBC molecular subtypes, which recently culminated into six consensus clusters; luminal papillary, luminal nonspecified, luminal unstable, stroma-rich, basal/squamous, and neuroendocrine-like [26,27,28,29,30,31,32,33]. Three molecular profiling studies suggested that patients with tumors identified by consensus subtyping as “basal/squamous” or “luminal nonspecified” are more likely to benefit from NAC as compared to patients with “stroma rich” tumors [26,30,33,34]. However, when evaluated in a prospective clinical trial, molecular subtyping of 161 patients was not able to significantly predict complete pathological response to NAC, nor did a cell line-based co-expression extrapolation (COXEN) prediction model (NCT02177695) [35]. Nonetheless, future analysis will demonstrate whether gene-expression profiling can predict for NAC-derived over-all survival benefit. 

Besides mRNA, also microRNA (miRNA) expression can be investigated in relation to patient treatment response. Thus far, several miRNAs have been associated with resistance to chemotherapy in vitro. mIR-196a-5p and mIR-294 have been shown to increase cisplatin resistance in bladder cancer cells [36,37]. Meanwhile, mIR-145 and miR-193b sensitize bladder cancer cell lines to cisplatin [38,39]. miRNA expression has also been used for molecular subtyping studies. Unsupervised clustering of 405 MIBC tumors identified a particular miRNA expression subtype characterized by high expression of the miR-200 that was associated with a relatively good prognosis [27]. In addition, miRNA expression patterns observed in MIBC were linked to gene regulatory networks controlling epithelial-mesenchymal transition (EMT) [27]. Likewise, expression of miRNA known to control EMT were also observed upregulated in tumor samples in a study comparing 23 matched normal and bladder tumor samples [40]. 

In short, neuroendocrine differentiation, somatic mutations in ERBB2 and several DNA-damage repair genes, as well as “basal/squamous” and “luminal nonspecified” molecular UC subtypes have been associated with favorable response to NAC. On the contrary, patients with either SCC or UC molecularly classified as “stroma-rich” are less likely to benefit from NAC. Clinical trials are currently evaluating the predictive value of both gene-expression profiling as well as somatic mutations in DNA-repair genes in respect to NAC-response. Meanwhile, miRNA expression has been associated with cisplatin response in vitro, warranting further investigations.

Clinical trials are now also assessing neoadjuvant ICI in patients refusing neoadjuvant cisplatin or whom are cisplatin ineligible. The Pure-01 phase II study investigated neoadjuvant administration of 3 cycles pembrolizumab in 119 patients, and demonstrated a pathological complete response (pCR) rate of 37% (NCT02736266) [41] The study reported pathological downstaging for eight out of nine patients with SCCs, whereas two out of three patients with lymphoepithelioma-like histological variants showed complete pT0 [41]. In addition, high tumor mutational burden (TMB) and high PD-L1 expression were associated with response to neoadjuvant pembrolizumab, as is similar to mUC [41]. In contrary, TMB and PD-L1 expression were not predictive of response to two cycles of neoadjuvant atezolizumab in the ABACUS phase II study (NCT02662309) [42]. The ABACUS trial reported a complete pathological response rate of 31%. The observed difference in complete pathological response (pCR) with PURE-01 could potentially be explained by administration of less cycles of ICI in the ABACUS trial. Interestingly, the ABACUS trial classified three out of four MIBC tumors as T cell inflamed, whereas only one out of four metastatic lesions classified as such [42,43]. This T cell inflamed UC phenotype was associated with response to ICI both in neoadjuvant and metastatic setting, which might indicate that neoadjuvant ICI might be suitable for selected patients [42,43]. Most recently, the NABUCCO trial evaluated neoadjuvant nivolumab combined with ipilimumab, a monoclonal antibody directed against cytotoxic T-lymphocyte-associated protein 4 (CTLA-4), in 24 patients with locally advanced MIBC (cT3-4aN0M0 or cT1-4aN1-3M0) and reported a pCR in 46% of the patients (NCT03387761) [44]. Interestingly, efficacy of nivolumab + ipilimumab was not associated with a pre-treatment T cell response [44].

In short, neoadjuvant ICI has been associated with complete pathological responses comparable to NAC, but overall survival benefit remains to be determined. Observed predictive value of PD-L1 expression, TMB and a T-cell inflamed phenotype warrant further research. Nonetheless, the observation that patients with SCC (whom do not respond to NAC) seem to respond to neoadjuvant ICI is especially interesting, warranting further investigations. 

Adjuvant targeted treatment modalities are also being evaluated in MIBC patients. Most recently, the IMvigor010 trial evaluated adjuvant atezolizumab in patients with residual disease after cystectomy, irrespective of NAC treatment [45]. However, no improvement of progression free survival (PFS) was demonstrated and no biomarkers predictive of benefit from adjuvant atezolizumab were identified [45]. FGFR inhibitors are also investigated in adjuvant setting, albeit only in patients with somatic FGFR3 aberrations. The PROOF-302 phase III trial randomizes MIBC patients with FGFR3 aberrations (N = 218) and either ≥ypT2 after NAC or ≥pT3 without prior NAC treatment at cystectomy for adjuvant infigratinib or placebo treatment (NCT04197986) [46]. Treatment schedule will consist of once daily oral dosing for two weeks, followed by one week off, for a total period of 52 weeks. Primary endpoint of this study is 1-year PFS.

In conclusion, no biomarkers have been identified that are able to robustly select MIBC patients for a specific treatment. Several histopathological and molecular biomarkers have been associated with patient response to either NAC or neoadjuvant ICI. However, those biomarkers remain to be validated in prospective clinical trials.

## 5. Upper Urinary Tract Urothelial Carcinoma (UTUC)

Standard treatment for patients with UTUC is a radical nephroureterectomy. NAC has been evaluated for UTUC patients and was associated with a survival benefit for patients with ≥pT2 UTUC [47]. Nevertheless, NAC-related renal toxicity hampers its usage. Furthermore, unreliability of clinical staging could lead to over-treatment of patients with low-grade disease [48]. Recently, the POUT randomized controlled trial reported a survival benefit for adjuvant gemcitabine + cisplatin (gem/cis) or gemcitabine + carboplatin (gem/carbo) treatment for patients with ≥pT2 and/or nodal disease after nephroureterectomy [49]. Meanwhile, the ongoing phase II URANUS trial randomized cisplatin-eligible patients with stage cT2-4 N0-1 UTUC to nephroureterectomy with either NAC or adjuvant chemotherapy and was expected to accrue in October 2020 (NCT02969083). Thus, chemotherapy will likely become standard therapy for patients with UTUC, either in adjuvant or neoadjuvant setting. Biomarkers predicting response to chemotherapy in UTUC have thus far not been evaluated.

Lastly, the previously mentioned PROOF-302 phase III trial is also randomizing UTUC patients (≥pT2 pN0-2 M0 stage tumors with somatic FGFR3-abbrations) to either adjuvant infigratinib or placebo treatment (NCT04197986). Thus, biomarker-directed therapy is currently not part of standard treatment of UTUC patients, although FGFR3-directed therapy is under investigation for selected patients.

## 6. Metastatic Urothelial Carcinoma (mUC)

First-line treatment for mUC patients is gem/cis which is associated with an ORR of 46% and is associated with a median survival of 14 months [50]. However, only 50% of mUC patients are cisplatin-eligible [2,51]. Cisplatin ineligibility is commonly defined as by meeting one of the following criteria: performance score (PS) > 1; glomerular filtration rate (GFR) ≤ 60 mL/min; grade ≥ 2 hearing loss; peripheral neuropathy; or New York Heart Association (NYHA) class III heart failure [51]. Cisplatin ineligible patients can be treated with gem/carbo instead, which is associated with a lower ORR of 36% and median survival of 9.3 months [52].

Platinum-relapsed patients can receive ICI as second-line treatment [2]. Pembrolizumab, atezolizumab and nivolumab (a PD-1 directed monoclonal antibody) are FDA-approved for treating platinum-relapsed or platinum-ineligible mUC patients, which is associated with objective response rates (ORR) of ~20% [53,54,55]. Although the median survival benefit for all treated patients is limited, it is important to emphasize that responses that do occur are often durable, as 68% of responses to pembrolizumab last over 12 months, while the median response duration to atezolizumab was 16 months [53,55]. Reliable biomarkers might prevent overtreatment of patients without survival benefit from ICI. PD-L1 expression on either tumor cells or a combination of tumor and immune cells was associated with response to second-line treatment with ICI, although responses were also observed in patients lacking PD-L1 expressing tumors [56,57,58]. Meanwhile, no predictive value of PD-L1 expression on tumor cells was observed in other studies [53,59]. Differences in predictive value of PD-L1 expression might be explained by either different drug efficacies and/or the application of different companion assays to detect and score PD-L1 expression. Use of a standardized diagnostic platform might provide as a solution to clarify observed differences. TMB has also been associated with response to second-line treatment with ICI, albeit not significantly [53,59].

Pembrolizumab and atezolizumab have also been investigated as first-line treatment option for cisplatin-ineligible patients. Pembrolizumab was associated with an ORR of 29%, whereas atezolizumab was associated with an ORR of 23% [57,60,61]. However, interim analyses of the ongoing KEYNOTE-361 phase III trial which randomizes cisplatin-ineligible patients to either gem/carbo or pembrolizumab treatment demonstrated inferior survival for patients with low PD-L1 expression treated with pembrolizumab monotherapy, compared to gem/carbo (NCT02853305) [62]. Therefore, first-line pembrolizumab has been FDA-approved only for cisplatin-ineligible patients with a PD-L1 combined positivity score (CPS) of ≥10%. Likewise, the FDA requires a PD-L1 positive tumor-infiltrating immune cells (IC) score of ≥5% for upfront treatment with atezolizumab, based on interim analysis of the IMvigor130 phase III trial (NCT02807636). Thus, first-line ICI seems only beneficial to cisplatin-ineligible patients with PD-L1 positive tumors.

FGFR-directed therapy has also been evaluated as second-line treatment for mUC patients. A single-arm phase II trial investigated erdafitinib in platinum-relapsed mUC patients and demonstrated an ORR of 34% for 99 patients with FGFR3 aberrations [63]. Based on those results, erdafitinib was granted accelerated FDA approval as second-line treatment for mUC patients with FGFR 2 or 3 alterations.

Infigratinib has been investigated in treatment-relapsed mUC patients with somatic activating FGFR3 mutations or fusions, and reported an ORR of 50% for progressed UTUC (N = 9) patients, while the ORR for MIBC patients (N = 59) was only 20% [64]. Different treatment responses might be explained by differences in specific FGFR3 driver mutation frequencies or tumor heterogeneity, as 50% of UTUC patients had an R248C mutation, compared to 12% of patients with UC of the bladder (UCB) (N = 59), while lower heterogeneity was reported UTUC patients compared to UCB patients [64]. Although no multivariate analysis was performed, and with the caveat of the small sample size, these results could indicate that patients with specific FGFR mutations might have different treatment responses to different FGFR inhibitors.

AZD4547 is an FGFR1-3 inhibitor that is being investigated in platinum-relapsed mUC patients carrying FGFR1-3 mutations in the BISCAY trial (NCT02546661) and MATCH trial (NCT02465060). FGFR1-4 inhibitor rogaratinib has demonstrated an ORR of 24% in 51 platinum-relapsed mUC patients selected based on high FGFR1-3 mRNA expression [65]. Interestingly, PIK3CA or RAS activating hotspot mutations, which act downstream of FGFR signaling, were associated with progressive disease [65].

Briefly, second-line FGFR-directed therapy proves beneficial for patients carrying somatic FGFR aberrations. Erdafitinib has received FDA approval for platinum-relapsed patients with FGFR 2/3 mutations or fusions. Preliminary evidence suggests that particular driver mutations might be associated with different treatment responses, and that PIK3CA or RAS activating mutations are associated with resistance to FGFR-inhibition.

Enfortumab vedotin is an antibody drug conjugate (ADC) composed of a nectin-4 directed antibody and cytotoxic agent vedotin. Nectin-4, which is strongly expressed in 60% of bladder tumors, is bound by enfortumab vedotin followed by internalization and degradation of the ADC leading to intracellular release of vedotin [66]. Adverse events often associated with enfortumab vedotin are peripheral neuropathy, fatigue, hyperglycemia and rash [67]. A phase I trial evaluated enfortumab vedotin in mUC patients who had progressive disease after platinum-based therapy and ICI (N = 128) and reported an ORR of 42% and a CR of 9% [68]. However, it was not reported whether nectin-4 expression or other biomarkers had predictive value for response to enfortumab vedotin.

The FDA granted accelerated approval of enfortumab vedotin as third-line treatment option for mUC patients who received prior platinum-based chemotherapy and ICI. Currently, a phase III RTC is investigating enfortumab vedotin as third-line treatment option compared to non-platinum-based chemotherapy of investigator’s choice (NCT03474107).

To conclude, first-line therapy for mUC patients is gem/cis, followed by second-line ICI with pembrolizumab for most patients, or second-line erdafitinib for patients with FGFR 2/3 aberrations. Cisplatin-ineligible patients with PD-L1 positive tumors as defined by drug specific companion diagnostic assays can be treated with either first-line pembrolizumab or atezolizumab. Gem/carbo remains the first-line treatment option for patients with without PD-L1 expressing tumors, although limited benefit should be expected for patients with both a PS > 1 and GFR ≤ 60 mL/min. Enfortumab vedotin has emerged as a third-line treatment option, and is currently being evaluated in compared to other third-line treatment options.

## 7. Renal Cancer

Renal cell carcinoma (RCC) is the 7th most common cancer in the Western world. In 2018, no less than 400,000 new cases and 175,000 deaths occurred worldwide [69]. Clear cell RCC (ccRCC) is the most common histological subtype of RCC (75%), followed by papillary RCC (type I and II, 10%), chromophobe RCC (5%) and several rare histological subtypes [70]. Apart from being the most common histological subtype, clear cell RCC (ccRCC) also more often progresses to metastatic disease as compared to the other histological subtypes. Therefore, most therapies have been focused primarily on ccRCC.

Due to the increased use of imaging modalities (Ultrasound, CT and MRI) in the last decades, more than half of RCCs are detected incidentally by imaging for other symptoms and diseases [71,72]. Nevertheless, approximately one third of ccRCC patients have synchronous metastases and one third of patients will develop metachronous metastatic disease during follow-up. Therefore, the 5-year overall survival (OS) of ccRCC patients in general is only 50% [73,74].

Standard therapy for localized and locally advanced RCC without metastases is surgery, either by partial nephrectomy (in ≤T1b tumors, up to 7cm) if feasible or by total nephrectomy [75,76]. Focal therapy, usually by radiofrequency ablation (RFA) or cryoablation is considered an acceptable alternative in ≤T1a tumors (up to 4cm) in elderly or comorbid patients [75,76]. Moreover, active surveillance, in order to prevent overtreatment, is shown to be relatively safe in ≤T1a tumors in elderly or comorbid patients with a low percentage of patients progressing to metastatic disease [73,77,78,79,80,81,82].

Currently, systemic targeted treatment is only approved and applied in advanced/metastatic RCC, as there is no evidence up to now for beneficial treatment (with VEGFR-TKIs) in the (neo-)adjuvant setting in localized and locally advanced RCC [83,84,85,86].

## 8. Adjuvant Targeted Treatment after Surgery in Non-Metastatic Renal Cell Carcinoma

Several phase III trials were conducted with VEGFR-TKIs (sunitinib, sorafenib, pazopanib, axitinib) in localized and locally advanced RCC (Table 2), none of which showed a clear survival benefit with VEGFR-TKI therapy [83,84,85,86]. Nevertheless, promising results of ICI in the metastatic setting have led to the evaluation of these compounds in the adjuvant setting for high-risk / locally advanced ccRCC (T2–4, N+): currently, four phase III trials are underway investigating ICIs (atezolizumab; nivolumab; pembrolizumab; nivolumab+ipilimumab) as an adjuvant treatment after surgery [87,88,89,90].

## 9. Vascular Endothelial Growth Factor Receptor—Tyrosine Kinase Inhibitors (VEGFR-TKIs)

Immunotherapy for metastatic RCC in the 1990s consisted of treatment with interferon alpha (IFN-α) or interleukin-2 (IL-2). IFN-α treatment was characterized by an incomplete response and a low response rate, with a median overall survival (OS) benefit of 2.5 months. Treatment with IL-2 was more potent and had a higher complete response rate (10–23%) as compared to IFN-α, although substantially more toxic [102,103,104].

New insights in the molecular pathways of RCC oncogenesis led to the development of targeted therapy. The von Hippel-Lindau (VHL) tumor-suppressor gene on the short arm of chromosome 3 is inactivated in up to 75% of ccRCC [105]. This causes an increased expression of vascular endothelial growth factor (VEGF), resulting in tumor neo-angiogenesis. Tyrosine kinase inhibitors (TKIs) were developed (e.g., sunitinib, sorafenib, pazopanib, axitinib, cabozantinib) to inhibit the VEGF family of receptors.

Sunitinib was the first VEGFR-TKI to be compared with standard of care. In the clinical trial of Motzer et al. a total of 750 patients with metastatic ccRCC and no prior treatment were randomized between 6-week cycles of sunitinib (orally) vs. IFN-α (subcutaneously 3x/week) (NCT00098657). A significant PFS was observed in the sunitinib arm of the trial (median 11 months vs. 5 months in the IFN-α arm) with a HR of 0.54 (95% CI 0.45–0.64) [91,92].

Similarly, a clinical trial was conducted with 435 treatment-naive patients with advanced or metastatic ccRCC randomized between treatment with pazopanib or placebo in a 2: 1 design [93]. Again, a significant improvement of the PFS was observed with pazopanib (Median PFS 11 months with pazopanib vs. 3 months with placebo) with a HR of 0.40 (95% CI 0.27–0.60) [93].

The efficacy and safety of sunitinib and pazopanib in the treatment-naïve setting were compared in the randomized COMPARZ study, including a total of 1110 patients with advanced or metastatic ccRCC (NCT00720941). No significant differences in PFS and OS were observed (median OS in the sunitinib arm 29 months vs. 28 months in the pazopanib arm), although the toxicity profile favored pazopanib [106].

Inclusion of patients with metastatic RCC for the phase III trials investigating VEGFR-TKIs was solely based on clinical characteristics and not biomarker-guided. The benefit of the VEGFR-TKIs extended across prognostic subgroups (based on these clinical characteristics) and was not correlated with specific biomarkers such as VEGFR-expression.

## 10. Second-Line Treatment: Mammalian Target of Rapamycin (mTOR) Inhibitors

Along with the identification of the VEGFR as a target for therapy, the mTOR-PI3K-AKT pathway was identified as a target. Mammalian target of rapamycin (mTOR) is activated/upregulated in up to 66% of metastatic RCC [107,108]. Efficacy of the mTOR inhibitor everolimus as a second-line treatment was assessed in the RECORD-1 study [99]. A total of 416 patients with metastatic ccRCC and prior treatment with one or two VEGFR-TKIs (sunitinib, sorafenib or both) were randomized with a 2: 1 ratio between treatment with everolimus 10mg once daily (orally) and placebo. A significant PFS benefit was observed in the treatment arm with everolimus (median PFS of 4.9 months vs. 1.9 months with placebo) with a corresponding HR of 0.33 (95% CI 0.25–0.43) [99].

## 11. Novel Second-Line Treatment in VEGFR-TKI-Resistant Disease

Cabozantinib is a compound, not only targeting the VEGFR but also MET and AXL, therefore overcoming resistance to VEGFR-targeted therapy [109]. Within the randomized METEOR study (NCT01865747), cabozantinib was compared with everolimus as a second-line treatment after VEGFR-TKI treatment in metastatic ccRCC [97]. A significant improvement of the PFS was observed with cabozantinib (Median PFS 7.4 months vs. 3.8 months with everolimus) with a corresponding HR of 0.58 (95% CI 0.45–0.75) [97].

As with bladder cancer, immune checkpoint inhibitors (ICI) were investigated in metastatic RCC, starting with the PD-1 inhibitor nivolumab. Nivolumab selectively blocks the interaction between PD-1, expressed on activated T-cells, and PD-L1 and PD-L2, expressed on immune cells and tumor cells. PD-L1 expression is shown to be associated with a poor prognosis in metastatic RCC [110,111,112]. PD-L1 expression could therefore be associated with improved response to nivolumab therapy in metastatic RCC. Similar to cabozantinib, nivolumab was compared with everolimus as a second-line treatment in metastatic ccRCC in the CheckMate-025 study (NCT01668784) [100]. A total of 821 patients with advanced or metastatic ccRCC and prior treatment with 1 or 2 VEGFR-TKIs were randomized between nivolumab infusion every 2 weeks (3 mg/kg IV) and everolimus 10mg orally daily. A significant overall survival (OS) benefit was seen in the nivolumab arm (median OS of 25 months vs. 20 months with everolimus) with a corresponding HR of 0.73 (95% CI 0.57–0.93) [100]. A total of 24% of patients in the CheckMate-025 study had PD-L1 expression on ≥1% of tumor-associated immune cells. PD-L1 expression was not associated with improved OS in response to nivolumab treatment [100].

These studies led to FDA and European Medicines Agency (EMA) approval as well as alteration of the European Association of Urology (EAU) guidelines on advanced RCC in 2016: after first-line treatment with a VEGFR-TKI (sunitinib, pazopanib), second-line treatment with either nivolumab or cabozantinib was advised in case of VEGFR-TKI-resistance rather than everolimus or a second VEGFR-TKI (axitinib, sorafenib) [113].

## 12. Immune Checkpoint Inhibition as First-Line Treatment in Metastatic (Clear Cell) Renal Cancer

After nivolumab showed superiority over everolimus as a second-line treatment in metastatic ccRCC, the efficacy of ICIs as a first-line treatment as compared to a VEGFR-TKI (sunitinib) was evaluated in three large randomized studies.

In the CheckMate-214 study (NCT02231749) a total of 1096 patients with metastatic RCC without prior treatment were randomized between sunitinib (standard of care) and a combination of nivolumab and ipilimumab (combination of ICIs approved for advanced melanoma) [94]. A significant increase of the overall survival (OS) was observed with nivolumab + ipilimumab treatment as compared to sunitinib alone in patients with intermediate- and poor-risk disease according to the International Metastatic Renal Cell Carcinoma Database Consortium (IMDC) criteria, with a HR of 0.63 (95% CI 0.44–0.89) [94]. The percentage of patients with quantifiable PDL-1 expression within the intermediate- and poor-risk disease group was 28%. Longer overall survival and higher objective respons rate were observed with nivolumab + ipilimumab as compared with sunitinib in the intermediate- and poor-risk group, although the magnitude of benefit was higher in the population with ≥1% PD-L1 expression [94].

Within the Keynote-426 study (NCT02853331) combination treatment of pembrolizumab (ICI) and axitinib (VEGFR-TKI) is compared with sunitinib [95]. A total of 861 patients with metastatic RCC without prior treatment were randomized in a 1:1 fashion between the treatment arms. Again, a superior overall survival (OS) was observed with combined treatment with pembrolizumab and axitinib as compared to sunitinib with a HR of 0.53 (95% CI 0.38–0.74) [95]. In contrast with the CheckMate-214 study, the benefit of pembrolizumab + axitinib as compared to sunitinib was observed across the IMDC risk groups, thus also in favorable risk disease. Furthermore, benefit of pembrolizumab + axitinib was observed regardless of PD-L1 expression [95].

Finally, within the randomized Javelin Renal 101 study (NCT02684006) the efficacy of the combination of avelumab (ICI) and axitinib (VEGFR-TKI) is compared with sunitinib [96]. A total of 886 patients with metastatic RCC without prior treatment were included, either with favorable, intermediate or poor-risk disease, of whom 560 patients (63%) had a PD-L1 positive tumor. A significant improve of the progression free survival (PFS), not overall survival (OS), was observed in the avelumab + axitinib arm with a corresponding HR of 0.69 (95% CI 0.56–0.84). A similar benefit in terms of PFS with avelumab + axitinib over sunitinib was observed in patients with PD-L1 expression (HR 0.61, 95% CI 0.47–0.79) [96].

These studies led to the FDA and EMA approval of these three treatment combinations in treatment-naïve metastatic RCC, regardless of PD-L1 expression status, as well as inclusion as first-line treatment in the updated EAU guidelines [75]: for patients in all IMDC risk-groups in case of pembrolizumab + axitinib and avelumab + axitinib), but limited to intermediate and poor-risk disease in case of nivolumab + ipilimumab.

microRNAs as biomarkers in renal cancer

Novel biomarkers are needed in order to improve patient-selection for (systemic) treatment in renal cell cancer (RCC) as well as evaluation of treatment response. MicroRNAs (miRNAs) participate in the pathogenesis of RCC and response of patients to therapeutic agents. Several circulatory and urinary miRNAs have promising properties as biomarkers in RCC [114]. For example, miR-144-3p has been linked with resistance to the VEGFR-TKI Sunitinib [115]. Further research is needed in order to establish suitable RCC miRNA panels.

## 13. Conclusions

The landscape of targeted therapy has changed in recent years for both BC and RCC, mainly due to the upcoming of immune checkpoint inhibitors. In metastatic bladder cancer, ICIs are currently approved as second line treatment in case of progression after chemotherapy, as well as first line treatment in PD-L1+ patients whom are not suitable for chemotherapy.

In renal cancer, ICIs are now considered first-line treatment in metastatic (clear cell) disease. In the (near) future there might be a place for ICIs as (neo-)adjuvant treatment for high-risk/locally advanced non-metastatic renal cancer. Despite high efficacy of ICI in a small proportion of bladder or renal cancer patients, the clinical application of ICI is hampered by limited efficacy among all treated patients, significant adverse events, and high costs. Future investigations should focus on identification of biomarkers for optimized patient selection.

## Figures and Tables

**Table 1:** Clinical trials investigating biomarker-guided treatment of bladder cancer.

Drug	Target	Trial Phase	Associated Biomarker	Patient Population	Status	Clinical Trial Information
**Treatment of HR BCG-unresponsive non-muscle invasive bladder cancer**						
Pembrolizumab	PD-1	II	-	HR BCG-unresponsive NMIBC	Recruiting	NCT02625961 (KEYNOTE-057) [6,7]
Atezolizumab	PD-L1	II	-	HR BCG-unresponsive NMIBC	Active, not recruiting	NCT02844816 (SWOG S1605) [8]
Pembrolizumab + BCG	PD-1	I	-	HR BCG-unresponsive NMIBC	Active, not recruiting	NCT02324582 (MARC)
Infigratinib	FGFR1-3	II	FGFR3 mutations or fusions		Closed due to low accrual	NCT02657486 [12]
Erdafitinib	FGFR1-4	II	FGFR3 mutations or fusions	HR BCG-unresponsive NMIBC	Recruiting	NCT04172675 [13]
**Perioperative treatment of MIBC**						
Active surveillance after AMVAC NAC	-	II	ATM, RB1, FANCC and/or ERCC2 mutations	MIBC	Recruiting	NCT02710734 (RETAIN)
Bladder sparing therapy after neoadjuvant gem/cis	-	II	Mutated DDR-genes	MIBC	NCT03609216	NCT03609216
NAC		II	Molecular subtyping/COXEN	MIBC	Active, not recruiting	NCT02177695 [35]
Neoadjuvant pembrolizumab	PD-1	II	-	MIBC	Recruiting	NCT02736266 (Pure-01) [41]
Neoadjuvant atezolizumab	PD-L1	II	-	MIBC	Active, not recruiting	NCT02662309 (ABACUS) [42]
Adjuvant infigratinib	FGFR1-3	III	FGFR3 aberrations	MIBC/UTUC	Recruiting	NCT04197986 (PROOF-302) [46]
**Treatment of metastatic urothelial cancer**						
First-line pembrolizumab	PD-1	III	PD-L1 CPS	Cisplatin-ineligible mUC	Active, not recruiting	NCT02853305 (KEYNOTE-361) [62]
First-line atezolizumab	PD-L1	III	PD-L1+ IC score	Cisplatin-ineligible mUC	Active, not recruiting	NCT02807636 (IMvigor130)
AZD4547	FGFR1-3	I	FGFR1-3 mutations	Platinum-relapsed mUC	Active, not recruiting	NCT02546661 (BISCAY)
AZD4547	FGFR1-3	II	FGFR1-3 mutations	Platinum-relapsed mUC	Recruiting	NCT02465060(MATCH)
Enfortumab vedotin	Nectin-4	III	-	Platinum + ICI relapsed mUC	Active, not recruiting	NCT03474107

AMVAC = accelerated MVAC; BCG = Bacillus Calmette-Guérin; COXEN = co-expression extrapolation; CPS = combined positivity score; DDR = DNA-damage response; HR = high risk; IC = immune cell; MIBC = muscle-invasive bladder cancer; mUC = metastasized urothelial carcinoma; MVAC = methotrexate, vinblastine, doxorubicin and cisplatin; NAC = Neoadjuvant chemotherapy; NMIBC = non-muscle invasive bladder cancer; PD-1 = programmed death receptor 1; PD-L1 = programmed death ligand 1.

**Table 2:** Phase III clinical trials investigating biomarker-guided treatment of renal cancer.

Drug	Target	Clinical Trial Information	Clinical Trial Design	Clinical Trial Results
**First line treatment for metastatic (clear cell) renal cell carcinoma**				
Sunitinib	VEGFRs	NCT00098657 [91,92]	RCT (*n* = 750; 1:1): Sunitinib vs. IFN-α	Superior PFS with Sunitinib (median 11 vs. 5 months), *HR* 0.54 (95% CI 0.45–0.64)
Pazopanib	VEGFRs	NCT00334282 [93]	RCT (*n* = 435; 2:1): Pazopanib vs. placebo	Superior PFS with Pazopanib (median 11 vs. 3 months), *HR* 0.40 (95% CI 0.27–0.60)
Nivolumab + Ipilimumab	PD-1	CheckMate-214 (NCT02231749) [94]	RCT (*n* = 1096; 1:1): Nivolumab + Ipilimumab vs. Sunitinib	Superior OS with Nivolumab + Ipilimumab, *HR* 0.63 (95% CI 0.44–0.89)
Pembrolizumab + Axitinib	PD-1 + VEGFRs	Keynote-426 (NCT02853331) [95]	RCT (*n* = 861; 1:1): Pembrolizumab + Axitinib vs. Sunitinib	Superior OS with Pembrolizumab + Axitinib, *HR* 0.53 (95% CI 0.38–0.74)
Avelumab + Axitinib	PD-1 + VEGFRs	Javelin Renal 101 (NCT02684006) [96]	RCT (*n* = 886; 1:1): Avelumab + Axitinib vs. Sunitinib	Superior PFS (not OS) with Avelumab + Axitinib, *HR* 0.69 (95% CI 0.56–0.84)
Atezolizumab + Bevacizumab	PDL-1	Immotion 151 (NCT02420821) [95]	RCT (*n* = 915; 1:1): Atezolizumab + Bevacizumab vs. Sunitinib	No superior PFS with Atezolizumab + Bevacizumab
Nivolumab + Ipilimumab + Cabozantinib	PD-1 + VEGFRs	COSMIC-313 (NCT03937219) [97]	RCT (targeted accrual *n* = 840): Nivolumab + Ipilimumab + Cabozantinib vs. Nivolumab + Ipilimumab + placebo	Trial ongoing, primary endpoint = PFS
**Second line treatment for metastatic (clear cell) renal cell carcinoma (after treatment with VEGFR-TKI)**				
Axitinib / Sorafenib	VEGFRs	AXIS (NCT00678392) [98]	RCT (*n* = 723; 1:1): Axitinib vs. Sorafenib	Superior PFS with Axitinib (median 7 vs. 5 months), *HR* 0.67 (95% CI 0.54–0.81)
Everolimus	mTOR	RECORD-1 (NCT00410124) [99]	RCT (*n* = 416; 2:1): Everolimus vs. placebo	Superior PFS with Everolimus (median 5 vs. 2 months), *HR* 0.33 (95% CI 0.25–0.43)
Cabozantinib	VEGFRs + MET + AXL	METEOR (NCT01865747) [97]	RCT (*n* = 658; 1:1): Cabozantinib vs. Everolimus	Superior PFS with Cabozantinib (median 7 vs. 4 months), *HR* 0.58 (95% CI 0.45–0.75)
Nivolumab	PD-1	CheckMate-025 (NCT01668784) [100]	RCT (*n* = 821; 1:1): Nivolumab vs. Everolimus	Superior OS with Nivolumab (median 25 vs. 20 months), *HR* 0.73 (95% CI 0.57–0.93)
**Adjuvant treatment (after surgery) for high risk non-metastatic (clear cell) renal cell carcinoma**				
Sunitinib	VEGFRs	S-TRAC (NCT00375674) [83]	RCT (*n* = 615; 1:1): Sunitinib vs. placebo	No superior OS with Sunitinib, *HR* 0.92 (95% CI 0.66–1.28)
Sunitinib/Sorafenib	VEGFRs	ASSURE (NCT00326898) [84]	RCT (*n* = 1943; 1:1:1): Sunitinib vs. Sorafenib vs. placebo	No differences in PFS and OS between the Sunitinib, Sorafenib and placebo arms
Sorafenib	VEGFRs	SORCE (NCT00492258) [101]	RCT (*n* = 1711; 1:1:1): Placebo vs. Sorafenib 1yr vs. Sorafenib 3yr	No differences in PFS and OS between both Sorafenib arms and the placebo arm
Pazopanib	VEGFRs	PROTECT (NCT01235962) [85]	RCT (*n* = 1135; 1:1): Pazopanib vs. placebo	No superior OS with Pazopanib, *HR* 0.82 (95% CI 0.62–1.07)
Axitinib	VEGFRs	ATLAS (NCT01599754) [86]	RCT (*n* = 724; 1:1): Axitinib vs. placebo	No superior PFS with Axitinib, *HR* 0.87 (95% CI 0.66–1.15)
Atezolizumab	PD-L1	IMmotion010 (NCT03024996) [87]	RCT (*n* = 778; 1:1): Atezolizumab vs. placebo	Trial ongoing, primary endpoint = PFS
Nivolumab	PD-1	PROSPER (NCT03055013) [88]	RCT (1:1, targeted accrual *n* = 766): Nivolumab vs. placebo	Trial ongoing, primary endpoint = PFS
Pembrolizumab	PD-1	Keynote-564 (NCT03142334) [89]	RCT (*n* = 950; 1:1): Pembrolizumab vs. placebo	Trial ongoing, primary endpoint = PFS
Nivolumab + Ipilimumab	PD-1	CheckMate-914 (NCT03138512) [90]	RCT (targeted accrual *n* = 1600): Nivolumab + Ipilimumab vs. Nivolumab vs. placebo	Trial ongoing, primary endpoint = PFS

VEGFR = vascular endothelial growth factor receptor; RCT = randomized controlled trial; PFS = progression free survival; OS = overall survival; HR = hazard ratio; CI = Confidence interval; PD-1 = programmed death receptor 1; PD-L1 = programmed death ligand 1; mTOR = mammalian target of Rapamycin.
